# Gender-Specific Moderating Role of Physical Fitness and Cardiovascular Risk Factors in the Relationship Between BMI and C-Reactive Protein: Cross-Sectional Study

**DOI:** 10.2196/76485

**Published:** 2025-08-27

**Authors:** Chunyuan Lai, Yunqing Liu, Yue Yan, Boli Cheng, Yakun Liu, Fanshu Yu, Chengyuan Hu, Liqiang Su, Fanghui Li

**Affiliations:** 1 School of Physical Education, Jiangxi Normal University, Nanchang, China; 2 Changzhou Sports Hospital, Affiliated Sports Hospital of Nanjing Normal University, Changzhou, China; 3 School of Sport Sciences, Nanjing Normal University, Xianlin Campus, Xianlin Street, Qixia District, Nanjing, Jiangsu Province, 210023, China, 86 18262609418

**Keywords:** BMI, C-reactive protein, physical fitness, prevention of cardiovascular diseases, gender differences

## Abstract

**Background:**

Physical fitness and cardiovascular risk factors may affect C-reactive protein (CRP) levels by influencing BMI. However, the extent to which these factors influence CRP through BMI remains unclear.

**Objective:**

This study aims to explore how indicators related to physical fitness and cardiovascular risk factors affect the relationship between BMI and CRP levels.

**Methods:**

This study selected 453 participants from 518 individuals at a sports hospital in Jiangsu Province, including 231 (51%) males and 222 (49%) females. Using interaction effect analyses, CRP served as the dependent variable and BMI as the independent variable. Physical fitness and cardiovascular risk factors (body morphology, blood pressure, blood lipids, and blood glucose) were categorized into quartiles from the lowest (Q1) to the highest (Q4) and treated as effect modifiers for interaction effect analyses. Sex-specific analyses were conducted to explore the influence of BMI on CRP across different genders, based on physical fitness and cardiovascular risk factor-related indicators.

**Results:**

BMI was positively associated with CRP (β=0.203, 95% CI 0.163‐0.243; *P*<.001). After adjusting for confounding factors, this association remained significant (β=0.206, 95% CI 0.164‐0.248; *P*<.001). In the stratified gender analysis, interaction effect analyses showed that in females, waist circumference (median 110.5, IQR 93‐128; β=0.342, 95% CI 0.232‐0.452; *P*<.001), hip circumference (median 120, IQR 104‐136; β=0.361, 95% CI 0.260‐0.462; *P*<.001), waist-hip ratio (median 1.08, IQR 0.9‐1.25; β=0.291, 95% CI 0.204‐0.378; *P*<.001), diastolic blood pressure (median 101, IQR 85‐117; β=0.344, 95% CI 0.252‐0.436; *P*<.001), triglycerides (median 1.41, IQR 1.13‐1.68; β=0.313, 95% CI 0.216‐0.409; *P*<.001), triglyceride-rich lipoprotein cholesterol (median 0.64, IQR 0.51‐0.76; β=0.365, 95% CI 0.249‐0.481; *P*<.001), 20‐49 years one-leg standing time with closed eyes (median 3, IQR 1‐5; β=0.371, 95% CI 0.276‐0.466; *P*<.001), and 20‐49 years grip strength (median 6.5, IQR 6‐7; β=0.369, 95% CI 0.270‐0.467; *P*<.001) significantly influenced the effect of BMI on CRP. In males, etotal cholesterol, glucose, one-leg standing time with closed eyes, sit-and-reach, and grip strength demonstrated trends in the effect of BMI on CRP (*P*>.05).

**Conclusions:**

This study demonstrates a positive correlation between BMI and CRP with gender-specific characteristics. In females, multiple body morphology, physiological, and physical fitness indicators significantly influence the effect of BMI on CRP, while some indicators in males also exhibit trends. These findings suggest the necessity of developing health management strategies tailored to different genders, particularly for female populations. Such strategies should consider body morphology indicators such as waist circumference and hip circumference, physiological indicators including blood pressure and blood lipids, and physical fitness metrics such as one-leg standing time with closed eyes. This comprehensive approach can better regulate the impact of BMI on CRP and promote overall health.

## Introduction

Cardiovascular disease (CVD) remains the leading cause of death globally, with the Global Burden of Disease Study revealing 612 million cases of CVD in 2021, accounting for 26.8% of all deaths worldwide [1]. Since 1990, the prevalence of unhealthy lifestyles among Chinese residents, combined with a large population at risk for CVD and accelerated population aging, has made CVD one of the most significant threats to public health in China. In the composition of disease-related deaths among urban and rural residents in China, CVD ranks first, accounting for 48.98% and 47.35% of deaths in rural and urban areas, respectively, in 2021. Among every 5 deaths, 2 are attributed to CVD [2]. Research indicates that obesity is a major risk factor for global severe morbidity and all-cause mortality, significantly increasing the risk of CVDs such as hypertension, type 2 diabetes, dyslipidemia, coronary heart disease, and myocardial infarction [3].

Currently, BMI is recognized as the standard metric for evaluating obesity and is closely associated with inflammation and the occurrence of multiple CVDs [4,5]. However, in recent years, the use of BMI as an indicator for assessing obesity and CVD has been questioned [6]. Some studies have pointed out that BMI overlooks important factors affecting health status, such as age, gender, and ethnicity, and thus cannot comprehensively measure an individual’s health level [7,8]. The American Heart Association stated in a scientific statement that BMI has limitations in predicting individual body fat [9]. Research has found that using BMI in conjunction with lipid metabolism indicators can more accurately predict type 2 diabetes [10]. Additionally, one study indicated that the waist-hip ratio (WHR) is more effective than BMI in predicting mortality [11]. However, other studies argue that BMI is a direct factor in the onset of various diseases and is an independent risk factor for inflammation [12,13].

Although BMI has a certain effect in evaluating inflammation, it is influenced by various factors such as physical fitness and cardiovascular risk factors. Studies indicate a subtle association between adult body morphology, fat distribution, and inflammatory manifestations. Some research has elucidated that inflammation is linked to lipid levels, specifically demonstrating that elevated plasma C-reactive protein (CRP) levels are often accompanied by increased concentrations of total cholesterol (TC), triglycerides (TG), low-density lipoprotein cholesterol (LDL-C), and triglyceride-rich lipoprotein cholesterol (TRL-C), as well as decreased concentrations of high-density lipoprotein cholesterol (HDL-C) [14,15]. Notably, inflammation has been identified as a significant driver of insulin resistance, metabolic syndrome, and type 2 diabetes, suggesting an inseparable relationship between inflammation and blood glucose levels [16]. Additionally, low levels of physical fitness are associated with elevated plasma CRP levels, while reduced inflammation levels are closely related to improved physical health, which may be linked to decreased BMI [17]. Based on the above evidence, this study considers physical fitness and cardiovascular risk factors as key factors, with body morphology, blood pressure, blood lipids, and blood glucose as indicators of cardiovascular risk factors.

In early research, the critical variable of gender was often overlooked, primarily due to the substantial underrepresentation of female participants in both preclinical and clinical studies [18,19]. The National Institutes of Health implemented a policy requiring all National Institutes of Health–funded research projects to include both male and female participants [18]. This policy has driven in-depth exploration of gender differences across various research fields, and obesity research is no exception. Currently, significant evidence demonstrates that the pathogenesis, risk factors, and clinical implications of obesity all exhibit substantial gender-specific differences [12,20,21]. Therefore, systematically investigating gender-specific outcomes in research is fully justified by scientific evidence and necessity.

Therefore, this study aims to investigate the gender differences in the relationship between BMI and CRP under the influence of physical fitness and multiple cardiovascular risk factors, while exploring more comprehensive evaluation methods and intervention strategies. The findings hold significant practical implications for the prevention and management of inflammation induced by obesity and its associated risks, providing a scientific basis for the widespread implementation of public health education and the precise prevention and treatment of CVDs.

## Methods

### Participants and Study Design

This cross-sectional study was conducted at a sports hospital in Changzhou City, Jiangsu Province, and 518 participants aged 20 years and older were recruited using convenience sampling. Data were collected between December 2022 and June 2023, and all participants underwent a systematic physical examination by a health care professional and signed an informed consent form. The examination included a physical examination, blood pressure and blood biochemical tests (including fasting blood glucose, blood lipids, and CRP). Based on the exclusion criteria, the following participants were excluded: age less than 20 years (n=15), long-term medication use (n=14), and lack of health screening data (n=36). After these exclusion criteria, the final effective sample size was 453.

### Inclusion and Exclusion Criteria

Since the proportion of individuals under 20 years old with CVD is relatively low, and there are many physiological and behavioral variables in minors that are difficult to control in the study, this study selected individuals aged 20 years and above as the study population [22-24].

Inclusion criteria are as follows: (1) age ≥20 years; (2) no acute inflammatory diseases; (3) presence of at least one cardiovascular risk factor (eg, hypertension, hyperlipidemia, hyperglycemia, smoking history, and obesity); (4) no psychological or consciousness disorders; and (5) complete baseline information, body morphology, blood pressure, blood lipids, blood glucose, and physical fitness–related health examination data.

Exclusion criteria are as follows: (1) individuals with mental disorders or severe intellectual disabilities; (2) pregnant or lactating females; (3) patients with a history of major cardiovascular events (eg, heart failure, myocardial infarction, unstable angina, and stroke); (4) diseases or treatments that may affect body composition and inflammatory status, such as corticosteroid use, advanced renal failure, bariatric surgery, severe fluid retention syndrome, and cancer; and (5) missing health examination data.

### Ethical Considerations

This study has obtained approval from the Biomedical Research Ethics Committee of Nanjing Normal University (NNU202310007). All participants provided informed consent prior to their involvement in the study, and they were explicitly informed of their right to withdraw at any point without any negative consequences. All data were coded to ensure the confidentiality of participant information, and additional measures, such as secure data storage and restricted access, were implemented to further protect privacy. No compensation was provided to the participants, as their involvement was entirely voluntary and based on their willingness to contribute to the research.

### Observation Indicators

The outcome measures in this study include CRP, body morphology, blood pressure, blood lipids, blood glucose, and physical fitness [25-27]. In 1999, Yudkin et al [28] proposed that obesity induces a systemic, persistent low-level chronic inflammatory state mediated by various inflammatory factors, based on the acute inflammation symptoms of “redness, swelling, heat, and pain.” Inflammation is generally assessed using inflammatory markers such as CRP and tumor necrosis factor-alpha [28]. Moreover, studies have shown that elevated CRP levels are an indicator of inflammation, a major risk factor for CVD [29-31]. Therefore, in this study, CRP was used as the inflammatory marker.

In addition, studies indicate a significant positive correlation between body morphology and CVD mortality, highlighting the importance of body morphology in CVD prevention [32,33]. Blood pressure, blood lipids, blood glucose, and inflammation are recognized as major CVD risk factors, and abnormalities in these indicators directly increase the risk of cardiovascular events [34]. Strong evidence demonstrates that improving physical fitness not only reduces the incidence of CVD events but also extends life span, underscoring the critical role of physical fitness in CVD prevention [35]. Additionally, recent research has found that cholesterol in TRL-C, as a product of their hydrolysis, is closely associated with diabetes and CVD [36]. Based on a comprehensive consideration of extensive research evidence and clinical practice, this study incorporates body morphology, blood pressure, blood lipids, blood glucose, and physical fitness as covariates to validate their roles in the effect of BMI on CRP.

Body morphology includes height, weight, waist circumference (WC), hip circumference (HC), WHR, and BMI. Blood pressure includes systolic blood pressure (SBP) and diastolic blood pressure (DBP). Blood lipids include TC, TG, TRL-C, LDL-C, and HDL-C. Blood glucose includes glucose (GLU) and glycated hemoglobin (HbA_1c_). Physical fitness includes one-leg standing time with closed eyes, sit-and-reach, and grip strength (see Multimedia Appendix 1 for physical fitness test methodology [37,38]), assessed based on the National Physical Fitness Test Standards Manual and Standards (Adults) and the National Physical Fitness Test Standards Manual (Elderly), published by the General Administration of Sport of China in 2023 [37]. The calculation method for TRL-C is as follows: when TG<4.5 mmol/L, LDL-C is calculated using the Friedewald formula (LDL-C = TC – HDL-C – TG/2.2); when TG≥4.5 mmol/L, LDL-C is directly measured; subsequently, the TRL-C concentration is calculated using the formula (TRL-C = TC – HDL-C – LDL-C) proposed by Varbo et al [36]. The indicators were divided into quartiles from lowest (Q1) to highest (Q4).

### Statistical Analysis

Data were processed and analyzed using R version 4.3.1 (The R Foundation for Statistical Computing) and SPSS version 26.0 (IBM Corp). For the basic characteristics of the study participants, participants were grouped and described by gender. Differences among groups in body morphology indicators, blood pressure, blood lipids, blood glucose, physical fitness, and CRP were compared. Continuous variables that follow a normal distribution are expressed as mean (SD), and group comparisons are performed using independent samples *t* tests. Continuous variables that do not follow a normal distribution are expressed as median (IQR), and group comparisons are performed using the Kruskal-Wallis test. If the Kruskal-Wallis test reveals significant differences, post hoc analysis is subsequently conducted to identify specific groups between which significant differences exist.

To verify whether BMI is an independent risk factor for inflammation, linear regression models with adjusted variables were used. In these models, CRP was set as the dependent variable, and BMI as the independent variable. Age, gender, smoking, and alcohol consumption were included as covariates for adjustment. The effects of BMI on CRP were observed both before and after adjustment to determine if BMI independently influences CRP. To address the issue of multiple comparisons, the Bonferroni correction method is applied in the interaction tests, adjusting the significance level to reduce the risk of false positives.

To assess the interaction effects of body morphology indicators, blood pressure, blood lipids, blood glucose, and physical fitness on the relationship between BMI and CRP, CRP was used as the dependent variable and BMI was set as the independent variable. The aforementioned covariates were divided into quartiles from lowest (Q1) to highest (Q4) to serve as interaction effect modifiers, and age, gender, smoking, and alcohol consumption were included as covariates for adjustment. This analysis aimed to determine whether there are interaction effects between the covariates divided into quartiles and BMI on CRP.

The study was stratified by gender into male and female groups for separate interaction effect analyses. Within each gender group, CRP was set as the dependent variable and BMI as the independent variable. The selected covariates were divided into quartiles from lowest (Q1) to highest (Q4) and used as interaction effect modifiers to analyze whether there are interaction effects between the covariates divided into quartiles and BMI on CRP in different genders, and age, smoking, and alcohol consumption were included as covariates for adjustment. Statistical significance was defined as *P*<.05, with *P*<.001 indicating highly significant.

## Results

### Description of Basic Characteristics of the Study Population

**Figure 1.**
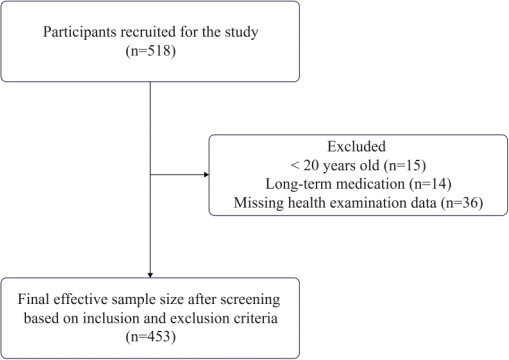
Participant flowchart.

As shown in Table 1 and Figure 1, a total of 518 participants were initially enrolled in the study and screened according to the inclusion and exclusion criteria, of which 453 participants including 231 males and 222 females were finally enrolled in this study. The mean age of females was 42.1 (SD  10.6) years, while that of males was 44.2 (SD 10.7) years. In the comparison between males and females, all other indicators showed statistically significant differences (*P*<.05) except for TC, HbA_1c_, and CRP.

**Table 1.** Basic characterization of the study population by gender grouping.

Indicator	Male (n=231)	Female (n=222)	*t* test (*df*)	*P* value
Age (years), mean (SD)	44.2 (10.7)	42.1 (10.6)	–2.06 (451)	.04
Height (cm), mean (SD)	172.8 (6.4)	160.6 (5.7)	–21.4 (451)	<.001
Weight (kg), mean (SD)	83.2 (14.8)	66.1 (13.5)	–12.9 (451)	<.001
WC^a^ (cm), mean (SD)	96.6 (10.4)	85.7 (11.9)	–10.4 (451)	<.001
HC^b^ (cm), mean (SD)	103.2 (7.8)	99.0 (9.1)	–5.22 (451)	<.001
WHR^c^ (cm), mean (SD)	0.93 (0.05)	0.86 (0.07)	–11.9 (451)	<.001
SBP^d^ (mm Hg), mean (SD)	134 (14)	123 (17)	–7.38 (451)	<.001
DBP^e^ (mm Hg), mean (SD)	86 (11)	78 (11)	–7.67 (451)	<.001
LDL-C^f^ (mmol/L), mean (SD)	1.58 (0.75)	1.39 (0.74)	–2.73 (451)	.007
HDL-C^g^ (mmol/L), mean (SD)	1.21 (0.30)	1.42 (0.35)	6.93 (451)	<.001
TG^h^ (mmol/L), median (IQR)	2.06 (1.26)	1.40 (1.1)	–6.01 (451)	<.001
TC^i^ (mmol/L), median (IQR)	5.02 (0.96)	4.88 (0.94)	–1.59 (451)	.11
TRL-C^j^ (mmol/L), mean (SD)	0.94 (0.61)	0.62 (0.48)	–6.19 (451)	<.001
HbA_1c_^k^ (%), median (IQR)	5.30 (0.70)	5.18 (0.64)	–1.87 (451)	.06
GLU^l^ (mmol/L), median (IQR)	5.95 (1.6)	5.53 (1.4)	–3.07 (451)	.002
One-leg standing time with closed eyes (seconds), mean (SD)	15.6 (19.1)	20.0 (22.5)	2.24 (451)	.03
Sit-and-reach (cm), mean (SD)	–2.39 (9.7)	3.54 (9.3)	6.66 (451)	<.001
Grip strength (kg), mean (SD)	40.8 (7.8)	23.8 (5.0)	–27.5 (451)	<.001
BMI (kg/m^2^), mean (SD)	27.76 (4.1)	25.6 (4.7)	–5.28 (451)	<.001
CRP^m^ (mg/L), mean (SD)	2.72 (2.3)	2.40 (2.1)	–1.57 (451)	.12

a WC: waist circumference.

b HC: hip circumference.

c WHR: waist-hip ratio.

d SBP: systolic blood pressure.

e DBP: diastolic blood pressure.

f LDL-C: low-density lipoprotein cholesterol.

g HDL-C: high-density lipoprotein cholesterol.

h TG: triglycerides.

i TC: total cholesterol.

j TRL-C: triglyceride-rich lipoprotein cholesterol.

k HbA_1c_: glycated hemoglobin.

l GLU: glucose.

m CRP: C-reactive protein.

### Relationship Between BMI and CRP

A linear regression analysis was conducted with CRP as the dependent variable and BMI as the independent variable. The results indicated that in the unadjusted model, each unit increase in BMI was associated with an increase in CRP concentration of 0.203 mg/L, and this effect was statistically significant (β=0.203, 95% CI 0.163‐0.243; *P*<.001). Similarly, after adjusting for age, gender, smoking, and alcohol consumption as confounding variables, the effect of BMI on CRP slightly increased to 0.206 mg/L, meaning that each unit increase in BMI was associated with an increase in CRP concentration of 0.206 mg/L, and this effect remained statistically significant (β=0.206, 95% CI 0.164‐0.248; *P*<.001; Table 2).

**Table 2.** Linear regression relationship between BMI and CRP^a^^,^^b^.

	Model 1^c^		Model 2^d^	
Variant	β (95% CI)	*P* value	β (95% CI)	*P* value
BMI	0.203 (0.163-0.243)	<.001	0.206 (0.164-0.248)	<.001

a CRP: C-reactive protein.

b CRP as the dependent variable and BMI as the independent variable.

c Model 1: unadjusted model.

d Model 2: adjusted for age, gender, smoking, and alcohol consumption.

### The Role of Cardiovascular Risk Factors in the Effect of BMI on CRP

Using CRP as the dependent variable and BMI as the independent variable, the adjustment for age, gender, smoking, and alcohol consumption had little effect on the β coefficient (Table 3).

**Table 3.** Interaction analysis of covariates (n=453).

Indicator	n (%)	Model 1^a^		*P* value	Model 2^b^		*P* value
		β (95% CI)	*P* value		β (95% CI)	*P* value	
WC^c^ (cm)				.08			.13
Q1	120 (26.5)	0.131 (–0.017 to 0.279)	.08		0.138 (–0.017 to 0.294)	.08	
Q2	115 (25.4)	0.162 (0.006 to 0.318)	.04		0.168 (0.003 to 0.333)	.047	
Q3	105 (23.2)	0.083 (–0.068 to 0.233)	.28		0.090 (–0.066 to 0.247)	.26	
Q4	113 (24.9)	0.285 (0.192 to 0.378)	<.001		0.285 (0.190 to 0.380)	<.001	
HC^d^ (cm)				.15			.32
Q1	131 (28.9)	0.161 (0.024 to 0.298)	.02		0.189 (0.044 to 0.333)	.011	
Q2	102 (22.5)	0.124 (–0.030 to 0.278)	.12		0.144 (–0.018 to 0.307)	.082	
Q3	112 (24.7)	0.199 (0.061 to 0.337)	.005		0.213 (0.072 to 0.355)	.003	
Q4	108 (23.8)	0.296 (0.209 to 0.384)	<.001		0.297 (0.209 to 0.385)	<.001	
WHR^e^ (cm)				.37			.37
Q1	115 (25.4)	0.123 (0.024 to 0.222)	.02		0.120 (0.019 to 0.221)	.02	
Q2	112 (24.7)	0.167 (0.048 to 0.285)	.006		0.162 (0.041 to 0.283)	.009	
Q3	112 (24.7)	0.171 (0.075 to 0.266)	<.001		0.161 (0.062 to 0.261)	.002	
Q4	114 (25.2)	0.230 (0.157 to 0.304)	<.001		0.225 (0.150 to 0.301)	<.001	
SBP^f^ (mm Hg)				.16			.2
Q1	122 (26.9)	0.094 (–0.010 to 0.198)	.08		0.103 (–0.004 to 0.209)	.059	
Q2	107 (23.6)	0.235 (0.140 to 0.329)	<.001		0.238 (0.141 to 0.334)	<.001	
Q3	116 (25.6)	0.227 (0.153 to 0.301)	<.001		0.228 (0.153 to 0.303)	<.001	
Q4	108 (23.8)	0.211 (0.134 to 0.289)	<.001		0.214 (0.134 to 0.295)	<.001	
DBP^g^ (mm Hg)				.31			.22
Q1	124 (27.4)	0.171 (0.090 to 0.252)	<.001		0.170 (0.088 to 0.251)	<.001	
Q2	108 (23.8)	0.149 (0.074 to 0.225)	.001		0.144 (0.067 to 0.221)	<.001	
Q3	112 (24.7)	0.236 (0.148 to 0.324)	<.001		0.248 (0.159 to 0.338)	<.001	
Q4	109 (24.1)	0.238 (0.152 to 0.324)	<.001		0.234 (0.147 to 0.322)	<.001	
LDL-C^h^ (mmol/L)				.9			.9
Q1	114 (25.2)	0.184 (0.113 to 0.256)	<.001		0.187 (0.114 to 0.261)	<.001	
Q2	114 (25.2)	0.220 (0.137 to 0.303)	<.001		0.224 (0.140 to 0.308)	<.001	
Q3	111 (24.5)	0.219 (0.136 to 0.301)	<.001		0.223 (0.139 to 0.306)	<.001	
Q4	114 (25.2)	0.201 (0.112 to 0.290)	<.001		0.206 (0.116 to 0.296)	<.001	
HDL-C^i^ (mmol/L)				.52			.54
Q1	115 (25.4)	0.225 (0.133 to 0.318)	<.001		0.219 (0.125 to 0.313)	<.001	
Q2	114 (25.2)	0.237 (0.151 to 0.323)	<.001		0.241 (0.155 to 0.328)	<.001	
Q3	113 (24.9)	0.155 (0.070 to 0.239)	<.001		0.156 (0.071 to 0.242)	<.001	
Q4	111 (24.5)	0.187 (0.114 to 0.259)	<.001		0.192 (0.117 to 0.267)	<.001	
TG^j^ (mmol/L)				.21			.28
Q1	115 (25.4)	0.124 (0.035 to 0.213)	.006		0.132 (0.042 to 0.223)	.004	
Q2	112 (24.7)	0.247 (0.165 to 0.328)	<.001		0.248 (0.164 to 0.331)	<.001	
Q3	113 (24.9)	0.223 (0.143 to 0.304)	<.001		0.222 (0.139 to 0.304)	<.001	
Q4	113 (24.9)	0.196 (0.115 to 0.278)	<.001		0.194 (0.112 to 0.277)	<.001	
TC^k^ (mmol/L)				.50			.47
Q1	114 (25.2)	0.158 (0.071 to 0.244)	<.001		0.162 (0.074 to 0.249)	<.001	
Q2	114 (25.2)	0.233 (0.142 to 0.324)	<.001		0.243 (0.151 to 0.335)	<.001	
Q3	113 (24.9)	0.187 (0.104 to 0.270)	<.001		0.186 (0.102 to 0.270)	<.001	
Q4	112 (24.7)	0.234 (0.164 to 0.303)	<.001		0.235 (0.163 to 0.307)	<.001	
TRL-C^l^ (mmol/L)				.18			.23
Q1	113 (24.9)	0.119 (0.030 to 0.208)	.009		0.128 (0.037 to 0.218)	.006	
Q2	114 (25.2)	0.249 (0.168 to 0.330)	<.001		0.250 (0.167 to 0.333)	<.001	
Q3	118 (26.0)	0.219 (0.134 to 0.303)	<.001		0.219 (0.133 to 0.306)	<.001	
Q4	108 (23.8)	0.203 (0.124 to 0.282)	<.001		0.199 (0.118 to 0.279)	<.001	
HbA_lc_^m^ (%)				.04			.06
Q1	120 (26.5)	0.130 (0.044 to 0.215)	.003		0.133 (0.045 to 0.221)	.003	
Q2	132 (29.1)	0.129 (0.053 to 0.206)	.001		0.119 (0.040 to 0.198)	.003	
Q3	104 (23.0)	0.256 (0.160 to 0.352)	<.001		0.244 (0.147 to 0.341)	<.001	
Q4	97 (21.4)	0.245 (0.173 to 0.317)	<.001		0.235 (0.162 to 0.309)	<.001	
GLU^n^ (mmol/L)				.13			.11
Q1	115 (25.4)	0.148 (0.059 to 0.237)	.001		0.141 (0.049 to 0.233)	.003	
Q2	112 (24.7)	0.212 (0.125 to 0.300)	<.001		0.217 (0.126 to 0.307)	<.001	
Q3	112 (24.7)	0.137 (0.053 to 0.220)	.001		0.129 (0.043 to 0.214)	.004	
Q4	114 (25.2)	0.252 (0.181 to 0.322)	<.001		0.246 (0.174 to 0.317)	<.001	

a Model 1: unadjusted model.

b Model 2: adjusted for age, gender, smoking, and alcohol consumption.

c WC: waist circumference.

d HC: hip circumference.

e WHR: waist-hip ratio.

f SBP: systolic blood pressure.

g DBP: diastolic blood pressure.

h LDL-C: low-density lipoprotein cholesterol.

i HDL-C: high-density lipoprotein cholesterol.

j TG: triglycerides.

k TC: total cholesterol.

l TRL-C: triglyceride-rich lipoprotein cholesterol.

m HbA_1c_: glycated hemoglobin.

n GLU: glucose.

In females, WC (median 110.5, IQR 93‐128; β=0.342, 95% CI 0.232‐0.452; *P*<.001), HC (median 120, IQR 104‐136; β=0.361, 95% CI 0.260‐0.462; *P*<.001), WHR (median 1.08, IQR 0.9‐1.25; β=0.291, 95% CI 0.204‐0.378; *P*<.001), DBP (median 101, IQR 85‐117; β=0.344, 95% CI 0.252‐0.436; *P*<.001), TG (median 1.41, IQR 1.13‐1.68; β=0.313, 95% CI 0.216‐0.409; *P*<.001), TRL-C (median 0.64, IQR 0.51‐0.76; β=0.365, 95% CI 0.249‐0.481; *P*<.001), 20‐49 years one-leg standing time with closed eyes (median 3, IQR 1‐5; β=0.371, 95% CI 0.276‐0.466; *P*<.001), and 20‐49 years grip strength (median 6.5, IQR 6‐7; β=0.369, 95% CI 0.270‐0.467; *P*<.001) significantly influenced the effect of BMI on CRP. Additionally, in males, TC, GLU, one-leg standing time with closed eyes, sit-and-reach, and grip strength demonstrated trends in influencing the effect of BMI on CRP (*P*>.05; Table 4). For interaction plots, refer to Figure S1 in Multimedia Appendix 2.

**Table 4.** Interaction analysis of covariates by gender. Model: adjusted for age, smoking, and alcohol consumption.

Indicator	Male (n=231)				Female (n=222)			
	Indicator, median (IQR)	n (%)	Model: β (95% CI); *P* value	*P* value	Indicator, median (IQR)	n (%)	Model: β (95% CI); *P* value	*P* value
WC^a^ (cm)				.86				.002
Q1	81.5 (73-90)	63 (27)	0.141 (–0.116 to 0.399); .28		69 (60-78)	57 (26)	–0.004 (–0.228 to 0.220); .97	
Q2	93 (90-96)	60 (26)	0.137 (–0.184 to 0.459); .4		80.5 (78-83)	55 (25)	0.113 (–0.110 to 0.337); .32	
Q3	99 (96-102)	50 (22)	0.168 (–0.191 to 0.528); .36		88 (83-93)	55 (25)	0.018 (–0.155 to 0.190); .84	
Q4	118 (102-134)	58 (25)	0.248 (0.091 to 0.404); .002		110.5 (93-128)	55 (24)	0.342 (0.232 to 0.452);<.001	
HC^b^ (cm)				.75				.003
Q1	93.5 (89-98)	66 (29)	0.326 (0.058 to 0.593); .02		82.8 (71.5‐94)	65 (30)	0.011 (–0.158 to 0.181); .9	
Q2	100 (98-102)	50 (21)	0.092 (–0.248 to 0.431); .6		96 (94-98)	52 (23)	0.132 (–0.079 to 0.342); .22	
Q3	104.5 (102-107)	60 (26)	0.215 (–0.043 to 0.473); .1		101 (98-104)	52 (23)	0.296 (0.132 to 0.461); .001	
Q4	122 (107-137)	55 (24)	0.238 (0.087 to 0.389); .002		120 (104-136)	53 (24)	0.361 (0.260 to 0.462);<.001	
WHR^c^ (cm)				.58				.045
Q1	0.85 (0.79‐0.9)	59 (25)	0.011 (–0.184 to 0.206); .91		0.75 (0.68‐0.82)	56 (25)	0.164 (0.057 to 0.271); .003	
Q2	0.92 (0.9‐0.93)	57 (25)	0.122 (–0.072 to 0.315); .22		0.84 (0.82‐0.86)	55 (25)	0.103 (–0.048 to 0.255); .18	
Q3	0.95 (0.93‐0.97)	57 (25)	0.165 (–0.022 to 0.353); .09		0.88 (0.86‐0.9)	55 (25)	0.130 (0.016 to 0.244); .03	
Q4	1.05 (0.97‐1.12)	58 (25)	0.160 (0.035 to 0.286); .01		1.08 (0.9‐1.25)	56 (25)	0.291 (0.204 to 0.378);<.001	
SBP^d^ (mm Hg)				.82				.02
Q1	109.5 (95-124)	58 (25)	0.109 (–0.082 to 0.300); .27		99.5 (87-112)	64 (29)	0.060 (–0.064 to 0.185); .34	
Q2	129 (124-134)	60 (26)	0.170 (0.018 to 0.321); .03		116 (112-120)	47 (21)	0.311 (0.192 to 0.430);<.001	
Q3	138.5 (134-143)	61 (26)	0.175 (0.064 to 0.287); .002		126 (120-132)	55 (25)	0.275 (0.170 to 0.379);<.001	
Q4	159 (143-175)	52 (23)	0.093 (–0.077 to 0.263); .28		153 (132-174)	56 (25)	0.264 (0.182 to 0.346);<.001	
DBP^e^ (mm Hg)				.08				.008
Q1	67.5 (57-78)	61 (26)	0.139 (0.014 to 0.263); .03		60 (50-70)	63 (28)	0.187 (0.083 to 0.291); .001	
Q2	81.5 (78-85)	59 (26)	0.144 (0.018 to 0.270); .03		73 (70-76)	49 (22)	0.121 (0.024 to 0.219); .02	
Q3	88.5 (85-92)	56 (24)	0.258 (0.118 to 0.398);<.001		81 (76-85)	56 (25)	0.264 (0.152 to 0.377); <.001	
Q4	108.5 (92-125)	55 (24)	–0.027 (–0.199 to 0.145); .76		101 (85-117)	54 (25)	0.344 (0.252 to 0.436);<.001	
LDL-C^f^ (mmol/L)				.77				.8
Q1	0.66 (0.34‐0.97)	58 (25)	0.126 (0.009 to 0.244); .04		0.55 (0.26‐0.84)	56 (25)	0.247 (0.155 to 0.340); <.001	
Q2	1.17 (0.97‐1.36)	58 (25)	0.221 (0.074 to 0.368); .004		1 (0.84‐1.16)	56 (25)	0.196 (0.101 to 0.290); <.001	
Q3	1.77 (1.36‐2.18)	57 (25)	0.187 (0.043 to 0.330); .01		1.51 (1.16‐1.86)	54 (25)	0.257 (0.157 to 0.356); <.001	
Q4	5.6 (2.18‐3.41)	58 (25)	0.163 (0.012 to 0.314); .04		2.71 (1.86‐3.56)	56 (25)	0.247 (0.137 to 0.357); <.001	
HDL-C^g^ (mmol/L)				.27				.26
Q1	0.76 (0.52‐1)	58 (25)	0.022 (–0.146 to 0.190); .8		0.93 (0.68‐1.18)	57 (26)	0.312 (0.209 to 0.416); <.001	
Q2	1.11 (1‐1.21)	60 (26)	0.194 (0.051 to 0.337); .008		1.29 (1.18‐1.40)	54 (24)	0.242 (0.139 to 0.346); <.001	
Q3	1.31 (1.21‐1.41)	58 (25)	0.121 (–0.027 to 0.269); .11		1.52 (1.40‐1.64)	55 (25)	0.192 (0.093 to 0.291); .002	
Q4	1.78 (1.41‐2.14)	55 (24)	0.203 (0.096 to 0.309);<.001		2.29 (1.64‐2.94)	56 (25)	0.160 (0.016 to 0.303); .03	
TG^h^ (mmol/L)				.27				.01
Q1	0.84 (0.39‐1.28)	58 (25)	0.158 (0.015 to 0.302); .03		0.49 (0.23‐0.74)	57 (26)	0.065 (–0.052 to 0.182); .28	
Q2	1.51 (1.28‐1.74)	58 (25)	0.249 (0.102 to 0.396); .001		0.94 (0.74‐1.13)	54 (24)	0.242 (0.140 to 0.345); <.001	
Q3	2.1 (1.74‐2.45)	58 (25)	0.053 (–0.086 to 0.191); .46		1.41 (1.13‐1.68)	55 (25)	0.313 (0.216 to 0.409); <.001	
Q4	3.88 (2.45‐5.31)	57 (25)	0.165 (0.041 to 0.289); .01		3.47 (1.68‐5.26)	56 (25)	0.256 (0.152 to 0.360); <.001	
TC^i^ (mmol/L)				.26				.8
Q1	3.31 (2.21‐4.41)	58 (25)	0.063 (–0.082 to 0.207); .39		3.28 (2.31‐425)	56 (25)	0.260 (0.154 to 0.367); <.001	
Q2	4.72 (4.41‐5.02)	58 (25)	0.113 (–0.067 to 0.292); .22		4.53 (4,25‐4.81)	56 (25)	0.266 (0.162 to 0.371); <.001	
Q3	5.32 (5.02‐5.61)	57 (25)	0.161 (0.015 to 0.306); .03		5.18 (4.81‐5.54)	56 (25)	0.204 (0.108 to 0.300); <.001	
Q4	7.06 (5.61‐8.51)	58 (25)	0.236 (0.124 to 0.347); <.001		6.87 (5.54‐8.19)	54 (25)	0.231 (0.134 to 0.328); <.001	
TRL-C^j^ (mmol/L)				.29				.002
Q1	0.38 (0.18‐0.58)	58 (25)	0.159 (0.016 to 0.303); .03		0.22 (0.1‐0.34)	55 (25)	0.044 (–0.073 to 0.161); .46	
Q2	0.69 (0.58‐0.79)	58 (25)	0.250 (0.103 to 0.397); .001		0.43 (0.34‐0.51)	56 (25)	0.255 (0.155 to 0.354); <.001	
Q3	0.95 (0.79‐1.11)	63 (27)	0.064 (–0.065 to 0.193); .33		0.64 (0.51‐0.76)	55 (25)	0.365 (0.249 to 0.481); <.001	
Q4	1.73 (1.11‐2.34)	52 (23)	0.165 (0.033 to 0.298); .015		1.43 (0.76‐2.1)	56 (25)	0.242 (0.151 to 0.334); <.001	
HbA_lc_^k^ (%)				.5				.06
Q1	4.6 (4.3‐4.9)	62 (27)	0.071 (–0.067 to 0.209); .31		4.4 (4‐4.8)	58 (26)	0.174 (0.048 to 0.300); .007	
Q2	5.1 (4.9‐5.2)	72 (31)	0.116 (–0.013 to 0.245); .08		5 (4.8‐5.1)	60 (27)	0.106 (0.007 to 0.205); .04	
Q3	5.4 (5.2‐5.5)	48 (21)	0.207 (0.044 to 0.371); .01		5.3 (5.1‐5.4)	56 (25)	0.272 (0.159 to 0.385);<.001	
Q4	7.9 (5.5‐10.3)	49 (21)	0.185 (0.054 to 0.315); .006		6.8 (5.4‐8.1)	48 (22)	0.257 (0.175 to 0.339);<.001	
GLU^l^ (mmol/L)				.12				.35
Q1	4.65 (3.99‐5.3)	59 (25)	0.042 (–0.101 to 0.185); .57		4.32 (3.82‐4.81)	56 (25)	0.174 (0.043 to 0.305); .01	
Q2	5.5 (5.3‐5.7)	57 (25)	0.198 (0.056 to 0.339); .007		5.11 (4.81‐5.41)	55 (25)	0.314 (0.181 to 0.447);<.001	
Q3	5.95 (5.7‐6.2)	57 (25)	0.069 (–0.081 to 0.219); .37		5.63 (5.41‐5.84)	55 (25)	0.182 (0.085 to 0.280);<.001	
Q4	9.35 (6.2‐12.5)	58 (25)	0.229 (0.109 to 0.349);<.001		8.92 (5.84‐12)	56 (25)	0.237 (0.154 to 0.321);<.001	
One-leg standing time with closed eyes (score)								
20‐49 y (male: n=159; female: n=162)				.37				.03
Q1	3 (1-5)	62 (27)	0.194 (0.075 to 0.314); .002		3 (1-5)	50 (23)	0.371 (0.276 to 0.466);<.001	
Q2	5.25 (5‐5.5)	32 (14)	0.162 (–0.021 to 0.344); .08		5.65 (5‐6.3)	31 (14)	0.252 (0.128 to 0.376); .001	
Q3	6.75 (5.5‐8)	38 (16)	0.002 (–0.185 to 0.189); .98		7.15 (6.3‐8)	49 (22)	0.187 (0.073 to 0.302); .002	
Q4	9 (8-10)	27 (12)	0.138 (–0.079 to 0.355); .22		9 (8-10)	32 (14)	0.167 (0.047 to 0.288); .007	
50‐59 y (male: n=50; female: n=47)				.37				.55
Q1	3 (1-5)	20 (9)	0.332 (–0.012 to 0.676); .07		3 (1-5)	13 (6)	–0.093 (–0.357 to 0.172); .5	
Q2	5.63 (5‐6.25)	7 (3)	0.152 (–0.260 to 0.564); .47		5.75 (5‐6.5)	11 (5)	–0.092 (–0.401 to 0.216); .56	
Q3	7.38 (6.25‐8.5)	12 (5)	0.188 (–0.366 to 0.741); .51		7.65 (6.5‐8.8)	11 (5)	0.043 (–0.393 to 0.478); .85	
Q4	9.25 (8.5‐10)	11 (5)	–0.104 (–0.552 to 0.344); .65		9.4 (8.8‐10)	12 (5)	0.132 (–0.142 to 0.407); .35	
60‐79 y (male: n=22; female: n=13)				.06				.2
Q1	3 (1-5)	7 (3.)	0.606 (0.111 to 1.101); .04		3 (1-5)	5 (2)	0.063 (–0.756 to 0.881); .89	
Q2	5.88 (5‐6.75)	3 (1)	0.632 (–0.615 to 1.880); .34		5.5 (5-6)	3 (1)	0.744 (–0.336 to 1.824); .24	
Q3	7.13 (6.75‐7.5)	9 (4)	0.127 (–0.240 to 0.494); .51		7 (6-8)	1 (1)	0.123 (–0.398 to 0.643); .66	
Q4	8.75 (7.5‐10)	3 (1)	–0.034 (–0.840 to 0.771); .94		8.8 (8‐9.5)	4 (2)	–0.005 (–0.687 to 0.676); .99	
Sit-and-reach (score)								
20‐49 y (male: n=159; female: n=162)				.17				.2
Q1	2 (1-3)	83 (36)	0.126 (0.018 to 0.234); .02		3 (1-5)	66 (30)	0.303 (0.225 to 0.381);<.001	
Q2	4 (3-5)	18 (8)	0.174 (–0.119 to 0.467); .25		5.25 (5‐5.5)	20 (9)	0.259 (0.125 to 0.394);<.001	
Q3	5.75 (5‐6.5)	26 (11)	0.289 (0.123 to 0.455); .001		6.25 (5.5‐7)	34 (15)	0.245 (0.110 to 0.379);<.001	
Q4	8.25 (6.5‐10)	32 (14)	–0.018 (–0.255 to 0.218); .88		8.5 (7-10)	42 (19)	0.142 (0.014 to 0.271); .03	
50‐59 y (male: n=50; female: n=47)				.56				.86
Q1	4.5 (1.5‐7.5)	17 (7)	0.247 (–0.053 to 0.547); .11		4.5 (1.5‐7.5)	13 (6)	–0.058 (–0.309 to 0.193); .66	
Q2	8.45 (7.5‐9.4)	13 (5)	0.085 (–0.354 to 0.524); .71		8.65 (7.5‐9.8)	14 (6)	–0.040 (–0.308 to 0.227); .77	
Q3	10.7 (9.4‐12)	11 (5)	0.501 (0.038 to 0.965); .04		10.7 (9.8‐11.6)	11 (5)	0.086 (–0.227 to 0.398); .59	
Q4	13.1 (12‐14.2)	9 (4)	0.158 (–0.619 to 0.935); .69		13.3 (11.6‐15)	9 (4)	0.069 (–0.363 to 0.501); .76	
60‐79 y (male: n=22; female: n=13)				.39				.69
Q1	3 (1-5)	7 (3)	0.451 (–0.162 to 1.065); .18		4..25 (3‐5.5)	3 (1)	0.171 (–0.621 to 0.964); .7	
Q2	5.4 (5‐5.8)	6 (3)	0.055 (–0.463 to 0.573); .84		6 (5.5‐6.5)	3 (1)	0.202 (–0.204 to 0.608); .37	
Q3	6.85 (5.8‐7.9)	3 (1)	0.070 (–0.618 to 0.758); .85		6.75 (6.5‐7)	6 (3)	–0.038 (–0.812 to 0.736); .93	
Q4	8.7 (7.9‐9.5)	6 (3)	0.188 (–0.345 to 0.721); .5		8 (7-9)	1 (1)	0.104 (–0.670 to 0.879); .8	
Grip strength (score)								
20‐49 y (male: n=159; female: n=162)				.74				.045
Q1	3 (1-5)	42 (18)	0.133 (–0.053 to 0.319); .16		3 (1-5)	46 (21)	0.190 (0.081 to 0.299);<.001	
Q2	5.75 (5‐6.5)	35 (15)	0.204 (0.024 to 0.383); .03		5.5 (5-6)	45 (20)	0.308 (0.196 to 0.420);<.001	
Q3	7.25 (6.5‐8)	49 (21)	0.182 (0.055 to 0.309); .006		6.5 (6-7)	51 (23)	0.369 (0.270 to 0.467);<.001	
Q4	9 (8-10)	33 (14)	0.080 (–0.100 to 0.259); .39		8.5 (7-10)	19 (9)	0.198 (0.074 to 0.321); .002	
50‐59 y (male: n=50; female: n=47)				.62				.68
Q1	4.9 (1.5‐8.3)	6 (3)	–0.015 (–0.533 to 0.503); .96		4.5 (1.5‐7.5)	19 (9)	0.063 (–0.240 to 0.366); .69	
Q2	9.1 (8.3‐9.8)	18 (8)	0.139 (–0.252 to 0.531); .49		7.9 (7.5‐8.3)	14 (6)	0.098 (–0.215 to 0.411); .54	
Q3	10.5 (9.8‐11.1)	13 (6)	0.155 (–0.286 to 0.595); .5		9.4 (8.3‐10.5)	5 (2)	0.144 (–0.278 to 0.565); .51	
Q4	13.1 (11.1‐15)	13 (6)	0.329 (–0.021 to 0.679); .07		12.4 (10.5‐14.2)	10 (5)	–0.066 (–0.292 to 0.160); .57	
60‐79 y (male: n=22; female: n=13)				.13				.53
Q1	5.65 (1.5‐9.8)	5 (2)	0.410 (–0.273 to 1.093); .26		7.9 (7.5‐8.3)	5 (2)	0.192 (–0.781 to 1.165); .72	
Q2	10.2 (9.8‐10.5)	7 (3)	0.001 (–0.668 to 0.671); .99		9.1 (8.3‐9.8)	3 (1)	0.379 (–0.108 to 0.865); .19	
Q3	10.8 (10.5‐11.1)	5 (2)	0.457 (–0.080 to 0.994); .12		11.7 (9.8‐13.5)	1 (1)	0.221 (–1.037 to 1.480); .75	
Q4	13.1 (11.1‐15)	5 (2)	–0.009 (–0.717 to 0.699); .98		13.9 (13.5‐14.2)	4 (1)	0.765 (–0.493 to 2.022); .29	

a WC: waist circumference.

b HC: hip circumference.

c WHR: waist-hip ratio.

d SBP: systolic blood pressure.

e DBP: diastolic blood pressure.

f LDL-C: low-density lipoprotein cholesterol.

g HDL-C: high-density lipoprotein cholesterol.

h TG: triglycerides.

i TC: total cholesterol.

j TRL-C: triglyceride-rich lipoprotein cholesterol.

k HbA_1c_: glycated hemoglobin.

l GLU: glucose.

## Discussion

### Principal Findings

The findings of this study indicate that BMI is an independent risk factor for CRP, and its association with CRP is significantly influenced by body morphology, blood pressure, blood lipids, blood glucose, and physical fitness levels. Notably, the impact of BMI on CRP exhibits gender-specific differences. In the female population, WC, HC, WHR, SBP, DBP, TG, TRL-C, one-leg standing time with closed eyes in the 20‐49 years age group, and grip strength are significantly correlated with the influence of BMI on CRP. In contrast, in the male population, although no significant differences were observed, analysis across different IQR intervals reveals that most cardiovascular risk factors exhibit variations in their modulating effects on the BMI-CRP association. These findings underscore the importance of gender, physical fitness, and multiple cardiovascular risk factors in the relationship between BMI and CRP, providing new perspectives for further research into the potential application value of these risk indicators in CVD prevention.

The interaction analysis stratified by gender revealed significant gender differences in the impact of BMI on CRP, with WC, HC, and WHR identified as important risk factors for increased CRP in females (*P*<.05). A study investigating the relationship between novel and traditional anthropometric measures and inflammation found that traditional body morphology indicators (such as weight, BMI, WC, and WHR) were positively associated with CRP in females [39]. This finding aligns with the results of the current study, further supporting the notion that WC, HC, and WHR can serve as significant indicators of cardiovascular risk in females. Additionally, another study demonstrated that trunk fat ratio was positively associated with hypersensitive C-reactive protein (hsCRP), while lower limb fat ratio was negatively associated with hsCRP, with this association being statistically significant only in females [40]. The discrepancy between these findings and those of the current study may stem from differences in sample characteristics, as the female participants in that study were in the initial stages of menopause, a period characterized by the redistribution of fat from lower limb subcutaneous adipose tissue to the abdominal region, resulting in a more proinflammatory fat distribution pattern. Consequently, in females, health interventions should particularly focus on the management of WC, HC, and WHR. Although the impact of BMI on CRP is less pronounced in males than in females, attention to BMI management remains crucial, especially for overweight and obese males.

In females, SBP and DBP significantly interact with the effect of BMI on CRP (*P*<.05). In the Female Health Study, higher levels of CRP were associated with incident hypertension in females aged 45 years or older [41]. However, in the Physician’s Health Study performed in male only, CRP was not associated with a higher risk of hypertension among individuals aged 40‐84 years [42]. Additionally, a cohort study revealed a gender-specific association between circulating inflammatory marker levels and the incidence of blood pressure and hypertension. The results showed that higher levels of inflammation were significantly associated with elevated SBP and DBP in females, but not in males [43]. These findings are consistent with the results of the study, which further provides novel evidence underscoring the importance of controlling blood pressure for the prevention and treatment of inflammation-related CVD in females.

The effect of BMI on CRP is influenced by certain blood lipid indicators. In females, TG and TRL-C significantly interact with the relationship between BMI and CRP (*P*<.05), whereas in males, TC poses a greater risk for increases in CRP. The National Health and Nutrition Examination Survey, a population-based study, revealed an association between the systemic immune-inflammation index and hyperlipidemia, further exploring gender differences in the threshold effects of hyperlipidemia [15]. However, this study did not analyze the gender-specific associations between specific blood lipid indicators and CRP or reveal a complex interaction effect among BMI, CRP, and dyslipidemia. In contrast, this study elucidates this effect and provides gender-specific insights for personalized health guidance. It was not only found that lower TG and TRL-C levels provide a certain protective effect for females, helping to mitigate the negative impact of BMI on CRP, but also indicated that in males, greater attention should be paid to the influence of TC levels on inflammatory responses, thereby offering more possibilities for personalized health guidance.

In obese populations, the accumulation of visceral and subcutaneous fat activates the immune system, triggering inflammatory responses and leading to elevated circulating levels of proinflammatory cytokines [44]. This chronic inflammatory environment alters insulin signaling, thereby exacerbating insulin resistance. Prolonged insulin resistance can lead to carcinogenesis, CVD, and type 2 diabetes [45]. Additionally, a large-scale cohort study demonstrated a positive correlation between CRP levels and the risk of developing type 2 diabetes, revealing gender differences [46]. These findings are consistent with the results of the study, which provides additional evidence through gender-stratified analysis of specific blood glucose indicators, offering deeper insights into the relationship between blood glucose and inflammation. Although statistical analysis results showed no significant interaction between GLU and CRP (*P*>.05), the results indicated that in the male population, there is a trend where GLU influences the effect of BMI on CRP to some extent. This suggests that maintaining appropriate GLU levels is important for mitigating the potential adverse effects of BMI on CRP in males.

Static exercises (one-leg standing time with closed eyes, sit-and-reach, and grip strength) demonstrate varying protective effects on the relationship between BMI and CRP across different age groups and genders. Among females aged 20‐49 years, one-leg standing time with closed eyes and grip strength were negatively associated with CRP (*P*<.05). In males, static exercises were negatively associated with CRP, except for grip strength, which was positively associated with CRP in the 50‐59 years age group. A cross-sectional study demonstrated that higher absolute strength levels in older individuals are associated with lower inflammation levels, playing a key role in reducing the risk of cardiovascular mortality [17]. However, this study only used grip strength tests to assess muscle strength. Another cross-sectional study found a significant correlation between good physical fitness levels and lower plasma proinflammatory marker levels in the older population. Additionally, through gender-specific analysis, it further confirmed that physical activity has a positive effect on reducing inflammation levels in females [47]. The above studies focused on the association between different intensities of physical activity and inflammation but did not delve into specific quantitative indicators of physical fitness. This study, by examining multiple specific indicators of physical fitness, not only provides a more comprehensive new perspective on the age and gender differences in the impact of static exercises on CRP levels but also demonstrates that high levels of static exercise ability effectively modulate the influence of BMI on CRP in males.

This study reveals significant gender differences in physical fitness and cardiovascular risk factors, indicating that BMI-related modulation of CRP may be more pronounced in females than in males. These findings are likely attributed to biological disparities in fat metabolism and hormone levels between genders. For instance, females tend to store lipids in subcutaneous fat, while males are more prone to visceral fat accumulation. Such distinctions may render females more susceptible to BMI-associated inflammatory responses [48]. Furthermore, gender-specific factors, including chromosomal variations, sex hormones, metabolic profiles, social determinants, and lifestyle elements, could also contribute to these differences [49,50]. Consequently, the regulatory role of gender-specific factors in physical fitness and cardiovascular risk warrants considerable attention.

In this study, despite the implementation of stringent inclusion criteria and adjustment for multiple covariates (eg, age, gender, smoking, and alcohol consumption), several limitations may influence the interpretation of the results. First, the cross-sectional design precludes the establishment of causal relationships, providing only associations between variables. Second, this study used convenience sampling to recruit healthy residents who underwent self-assessments at sports hospitals as research subjects, potentially introducing selection bias, as individuals with better health status or higher health awareness may be more inclined to participate, thereby limiting the generalizability of the findings. Additionally, although gender-stratified interaction tests were conducted, potential confounding factors (eg, dietary habits and psychological stress) may not have been fully controlled, potentially affecting the interpretation of the relationship between BMI and CRP. Finally, despite comprehensive assessments (eg, physical examinations, blood biochemical tests, and imaging studies), unmeasured biomarkers or environmental factors could still exert potential influences on the results. Despite these limitations, the large sample size, rigorous standards, multivariable adjustments, and standardized procedures minimized bias and enhanced the reliability of the findings. Future studies should adopt longitudinal designs and incorporate additional potential confounders to further validate and extend the results of this study. This study exhibits several notable strengths. First, the adoption of a multidimensional and multilevel analytical framework provides an in-depth exploration of how physical fitness and cardiovascular risk factors influence CRP through BMI, offering novel perspectives and theoretical foundations for research in this field. Second, the implementation of gender-specific analytical methods reveals significant gender differences in the impact of BMI on CRP, which holds substantial practical implications for improving clinical practice and informing policy development.

### Limitations

This study also presents several limitations. First, the use of cross-sectional data precludes the inference of causal relationships between variables. The analytical results merely reflect associations between variables, and future studies should consider using longitudinal or experimental designs to further explore potential causal mechanisms. Second, the use of convenience sampling of participants recruited from sports hospitals for cross-sectional surveys limits the generalizability of the results and, as with most cross-sectional studies, there may be a selection bias that may render the sample unrepresentative, but we have tried to control bias as much as possible by clarifying the inclusion and exclusion criteria, tightly controlling the stage of data collection to improve the representativeness of the sample and the trustworthiness of the study, and so on. Subsequent studies may mitigate these biases by expanding the sampling scope, conducting multistage sampling, and others, to provide wider generalization value.

### Conclusions

BMI exerts a significant influence on CRP, with distinct cardiovascular risk factors, including body morphology, blood pressure, blood lipids, and blood glucose, and physical fitness modulating the BMI-CRP relationship differently between genders. Specifically, in females, body morphology, physiological parameters, and physical fitness significantly influence the relationship between BMI and CRP. In males, certain indicators also show similar trends, but the extent of the influence differs by gender. Therefore, when managing the BMI-CRP relationship in the female population, high-risk cardiovascular risk factors should be considered in combination with age in order to optimize intervention strategies. This finding provides an important scientific basis for the development of gender-specific health management strategies, which is of great clinical significance for reducing CVD risk and improving health outcomes.

## Supplementary material

10.2196/76485 Multimedia Appendix 1 Physical fitness test methodology.

10.2196/76485 Multimedia Appendix 2 Interaction plots and interaction analysis of covariates.

## Abbreviations

CRP: C-reactive protein
CVD: cardiovascular disease
DBP: diastolic blood pressure
GLU: glucose
HbA_1c_: glycated hemoglobin
HC: hip circumference
HDL-C: high-density lipoprotein cholesterol
hsCRP: hypersensitive C-reactive protein
LDL-C: low-density lipoprotein cholesterol
SBP: systolic blood pressure
TC: total cholesterol
TG: triglycerides
TRL-C: triglyceride-rich lipoprotein cholesterol
WC: waist circumference
WHR: waist-hip ratio
