# Supplementary material for: Gender-Specific Moderating Role of Physical Fitness and Cardiovascular Risk Factors in the Relationship Between BMI and C-Reactive Protein: Cross-Sectional Study
Source: JMIR Public Health Surveill. 2025 Aug 27;11:e76485. doi: 10.2196/76485 (PMC12385609; doi:10.2196/76485)
Supplement: Multimedia Appendix 1 [file publichealth-v11-e76485-s001.docx]

# Physical fitness test methodology

According to the National Physical Fitness Measurement Standard (2023 Revision) [37,38], the testing methods for physical fitness indicators are as follows:

(1) Grip Strength

Testing Instrument: Grip strength dynamometer (HK-6800-WL).

Instrument Components: One main unit and one grip force sensor.

Testing Procedure: The tester ensures the instrument is in working condition, with the main unit displaying a value of "0.0 kg."

Before testing, the subject adjusts the grip distance to a comfortable position using the dominant hand by turning the grip distance adjustment wheel, while the other hand holds the upper and lower handles.

During the test, the subject stands upright with feet naturally apart, shoulder-width apart, arms hanging diagonally, palms facing inward, and exerts maximum force to grip the upper and lower handles. The test is conducted twice consecutively, with the main unit automatically recording and saving the best result, which is then uploaded to an IC card (RF card) for storage. Grip strength values are recorded in kilograms, accurate to one decimal place.

(2) Sit-and-Reach Test

Testing Instrument: Sit-and-reach test apparatus(HK-6800-TQ).

Instrument Components: One main unit, one sit-and-reach sensor, one sit-and-reach board, and one leg strap.

Testing Procedure: The tester ensures the instrument is in working condition, with the cursor reset and the main unit displaying a value of "-22.0 cm."

The subject sits barefoot on the board, facing the apparatus, with legs extended forward, heels together, and the entire sole of the foot pressed against the apparatus's backplate, toes naturally apart. The tester adjusts the guide rail height to align the subject's toes with the lower edge of the cursor and secures the leg strap.

During the test, the subject extends both hands forward with palms facing downward, keeps the knees straight, and bends the torso forward. The subject uses the fingertips of both middle fingers to push the cursor smoothly forward until no further movement is possible. The test is conducted twice consecutively, with the main unit automatically recording and saving the best result, which is then uploaded to an IC card (RF card) for storage. Sit-and-reach values are recorded in centimeters, accurate to one decimal place.

(3) One-Leg Stand with Eyes Closed

Testing Instrument: One-leg stand test apparatus(HK-6800-ZL).

Instrument Components: One main unit and one one-leg stand sensor.

Testing Procedure: The tester ensures the instrument is in working condition, with the main unit displaying "0.0 s."

The subject steps onto the one-leg stand sensor with both feet, placing the dominant supporting foot on the central platform and the other foot on the side platform. The display shows "0," and a buzzer sounds. The subject closes their eyes, lifts the non-supporting foot, and the buzzer stops, initiating the timer. When the supporting foot moves or the non-supporting foot touches the ground, the buzzer sounds again, indicating the end of the test, and the display shows the test value. The test is conducted twice consecutively. After the first test, the subject steps off the sensor, and the main unit resets to "0.0 s" before the second test begins. The main unit automatically records and saves the best result, which is then uploaded to an IC card (RF card) for storage. One-leg stand values are recorded in seconds, without decimal places.
