# Supplementary material for: Gender-Specific Moderating Role of Physical Fitness and Cardiovascular Risk Factors in the Relationship Between BMI and C-Reactive Protein: Cross-Sectional Study
Source: JMIR Public Health Surveill. 2025 Aug 27;11:e76485. doi: 10.2196/76485 (PMC12385609; doi:10.2196/76485)
Supplement: Multimedia Appendix 2 [file publichealth-v11-e76485-s002.docx]

**Table S1.** Interaction analysis of covariates.

| Indicator | n（%） | Model 1 β (95%CI) *P* value | *P* value | Model 2 β (95%CI) *P* value | *P* value |
| --- | --- | --- | --- | --- | --- |
| One-leg standing time with closed eyes/s | 453 |  |  |  |  |
| 2049 | 321 |  | .129 |  | .174 |
| Q1 | 112 (24.7%) | 0.276 (0.201, 0.351) <.001 |  | 0.271 (0.194, 0.349) <.001 |  |
| Q2 | 63 (13.9%) | 0.214 (0.110, 0.319) <.001 |  | 0.211 (0.104, 0.318) <.001 |  |
| Q3 | 87 (19.2%) | 0.141 (0.046, 0.236) .004 |  | 0.142 (0.044, 0.240) .005 |  |
| Q4 | 59 (13.0%) | 0.168 (0.056, 0.280) .004 |  | 0.170 (0.056, 0.238) .004 |  |
| 5059 | 97 |  | .937 |  | .916 |
| Q1 | 33 (7.3%) | 0.141 (–0.064, 0.346) .182 |  | 0.147 (–0.064, 0.359) .176 |  |
| Q2 | 18 (4.0%) | 0.067 (–0.155, 0.288) .558 |  | 0.055 (–0.188, 0.299) .657 |  |
| Q3 | 23 (5.1%) | 0.126 (–0.165, 0.416) .400 |  | 0.086 (–0.228, 0.399) .594 |  |
| Q4 | 23 (5.1%) | 0.066 (–0.132, 0.263) .518 |  | 0.060 (–0.154, 0.275) .583 |  |
| 6079 | 35 |  | .759 |  | .529 |
| Q1 | 12 (2.6%) | 0.207 (–0.195, 0.609) .321 |  | 0.330 (–0.072, 0.732) .121 |  |
| Q2 | 6 (1.3%) | 0.381 (–0.227, 0.988) .230 |  | 0.509 (–0.097, 1.116) .113 |  |
| Q3 | 10 (2.2%) | 0.189 (–0.008, 0.385) .071 |  | 0.251 (0.023, 0.478) .041 |  |
| Q4 | 7 (1.5%) | 0.044 (–0.340, 0.429) .822 |  | 0.106 (–0.277, 0.488) .593 |  |
| Sit-and-reach /cm | 453 |  |  |  |  |
| 2049 | 321 |  | .092 |  | .128 |
| Q1 | 149 (32.9%) | 0.221 (0.156, 0.285) <.001 |  | 0.216 (0.149, 0.283) <.001 |  |
| Q2 | 38 (8.4%) | 0.228 (0.093, 0.364) .001 |  | 0.227 (0.090, 0.363) .001 |  |
| Q3 | 60 (13.2%) | 0.310 (0.206, 0.415) <.001 |  | 0.303 (0.197, 0.410) <.001 |  |
| Q4 | 74 (16.3%) | 0.104 (–0.018, 0.227) .095 |  | 0.108 (–0.015, 0.232) .087 |  |
| 5059 | 97 |  | .462 |  | .407 |
| Q1 | 30 (6.6%) | 0.099 (–0.070, 0.268) .254 |  | 0.107 (–0.075, 0.288) .252 |  |
| Q2 | 27 (6.0%) | 0.016 (–0.172, 0.204) .871 |  | –0.005 (–0.209, 0.200) .964 |  |
| Q3 | 22 (4.9%) | 0.254 (0.016, 0.492) .039 |  | 0.263 (0.005, 0.522) .049 |  |
| Q4 | 18 (4.0%) | 0.081 (–0.303, 0.464) .681 |  | 0.098 (–0.303, 0.498) .634 |  |
| 6079 | 35 |  | .527 |  | .708 |
| Q1 | 10 (2.2%) | 0.254 (–0.115, 0.623) .189 |  | 0.278 (–0.118, 0.673) .182 |  |
| Q2 | 9 (2.0%) | 0.201 (–0.010, 0.412) .073 |  | 0.171 (–0.053, 0.395) .148 |  |
| Q3 | 9 (2.0%) | –0.046 (–0.409, 0.317) .805 |  | 0.021 (–0.361, 0.404) .915 |  |
| Q4 | 7 (1.5%) | 0.068 (–0.386, 0.523) .770 |  | 0.135 (–0.421, 0.691) .639 |  |
| Grip strength/kg | 453 |  |  |  |  |
| 2049 | 321 |  | .393 |  | .353 |
| Q1 | 88 (19.4%) | 0.219 (0.122, 0.316) <.001 |  | 0.211 (0.112, 0.311) <.001 |  |
| Q2 | 80 (17.7%) | 0.233 (0.135, 0.332) <.001 |  | 0.233 (0.133, 0.333) <.001 |  |
| Q3 | 100 (22.1%) | 0.271 (0.193, 0.348) <.001 |  | 0.268 (0.189, 0.348) <.001 |  |
| Q4 | 52 (11.5%) | 0.156 (0.048, 0.263) .005 |  | 0.148 (0.038, 0.257) .009 |  |
| 5059 | 97 |  | .836 |  | .863 |
| Q1 | 25 (5.5%) | –0.004 (–0.261, 0.252) .975 |  | 0.004 (–0.264, 0.271) .979 |  |
| Q2 | 32 (7.1%) | 0.100 (–0.112, 0.312) .356 |  | 0.101 (–0.132, 0.333) .399 |  |
| Q3 | 18 (4.0%) | 0.137 (–0.151, 0.425) .354 |  | 0.151 (–0.148, 0.449) .324 |  |
| Q4 | 23 (5.1%) | 0.129 (–0.054, 0.312) .170 |  | 0.117 (–0.073, 0.308) .230 |  |
| 6079 | 35 |  | .618 |  | .750 |
| Q1 | 10 (2.2%) | 0.199 (–0.223, 0.621) .364 |  | 0.345 (–0.134, 0.823) .172 |  |
| Q2 | 10 (2.2%) | 0.228 (0.020, 0.436) .041 |  | 0.219 (–0.012, 0.451) .076 |  |
| Q3 | 6 (1.3%) | 0.112 (–0.221, 0.446) .514 |  | 0.334 (–0.075, 0.744) .123 |  |
| Q4 | 9 (2.0%) | –0.058 (–0.507, 0.392) .804 |  | 0.112 (–0.400, 0.623) .673 |  |

Note: CRP as the dependent variable; BMI as the independent variable. Data in the table are presented as β (95% CI) P-values.

Model 1: unadjusted model, Model 2: adjusted for age, gender, smoking, drinking.


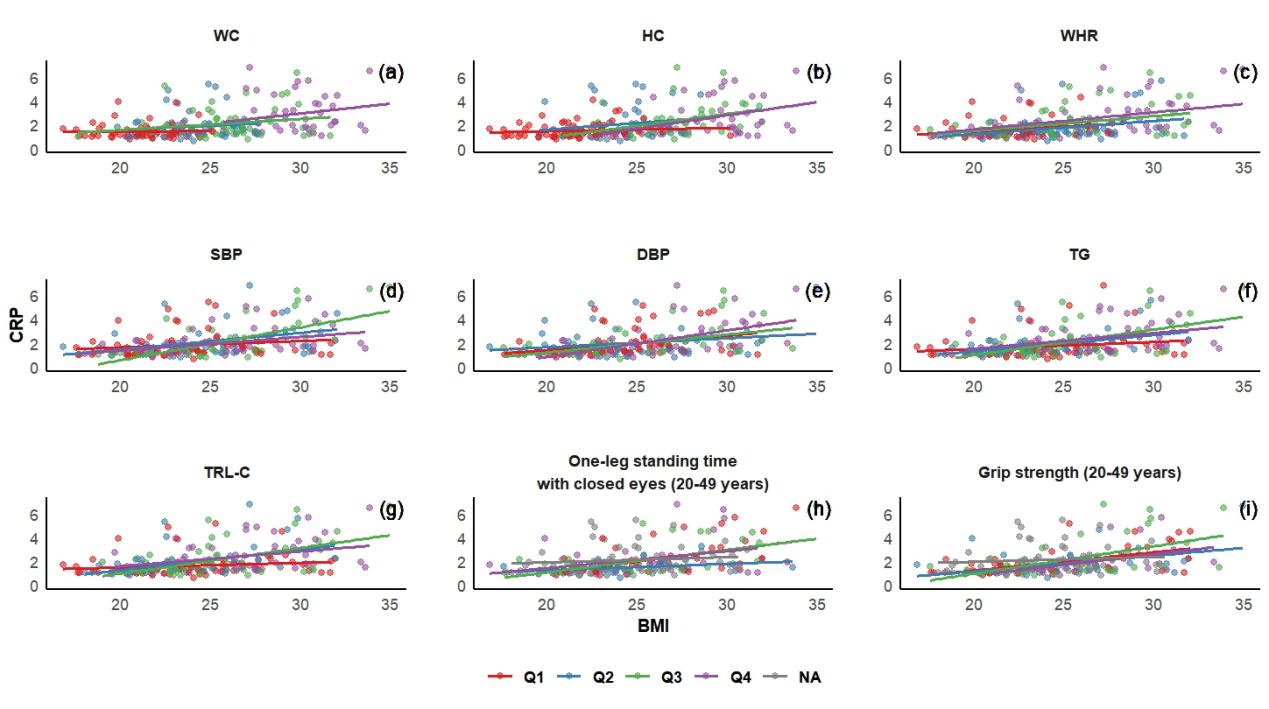


**Figure S1.** Interaction plots.
